# 2-Sulfanylidene-1,3-dithiolo[4,5-*b*]naphtho­[2,3-*e*][1,4]dithiine-5,10-dione

**DOI:** 10.1107/S1600536811039079

**Published:** 2011-10-05

**Authors:** Miguel Angel Méndez-Rojas, Sylvain Bernès, Aarón Pérez-Benítez, María Fernanda Romero Zarazúa, Adrián Castellanos-Uribe

**Affiliations:** a Departamento de Ciencias Químico-Biológicas, Universidad de las Américas Puebla, ExHda. de Sta. Catarina Mártir, 72820 San Andrés Cholula, Pue., Mexico; b DEP Facultad de Ciencias Químicas, UANL, Guerrero y Progreso S/N, Col. Treviño, 64570 Monterrey, N.L., Mexico; c Facultad de Ciencias Químicas, Benemérita Universidad Autónoma de Puebla, 14 Sur y av. San Claudio, Col. San Manuel, 72570 Puebla, Pue., Mexico

## Abstract

The title mol­ecule, C_13_H_4_O_2_S_5_, is folded by 47.83 (6)° along the S⋯S vector of the [1,4]dithiine six-membered ring, with the naphtho­quinone and [1,3]dithiole-2-thione moieties being nearly planar [largest deviations from least-squares planes = 0.028 (2) and 0.016 (1) Å, respectively]. This boat conformation is close to that observed in the analogous compound [Mendez-Rojas *et al.* (2001). *J. Chem. Crystallogr.* **31**, 17–28] including a 2-oxo group [folding angle: 42.3 (1)° at 213 (2) K]. Both compounds are indeed isomorphous, and the small difference in the folding angle probably results from the involvement of the thioxo group of the title compound in inter­molecular S⋯S contacts [3.5761 (13) Å]. In the crystal structure, mol­ecules are stacked in the [100] direction, with dithiole rings making π–π inter­actions. In a stack, alternating short and long separations are observed between the centroids of dithiole rings, 3.5254 (17) and 4.7010 (18) Å.

## Related literature

For general background to sulfur-containing heterocycles in organic conductors, see: Wudl (1984 ▶); Jérome (2007 ▶). For dithiine derivatives and their redox behavior, see: Hayakawa *et al.* (1982 ▶); Kao *et al.* (1985 ▶); Kniess & Mayer (1996 ▶); Brisse *et al.* (2000 ▶); Mendez-Rojas *et al.* (2001 ▶). For the synthesis of the precursor of the title dithiine, see: Wang *et al.* (1998 ▶).
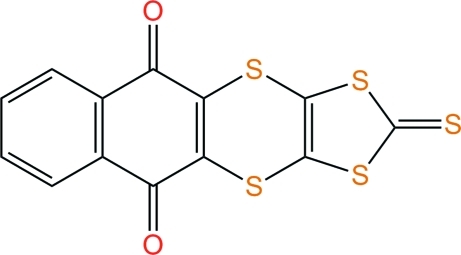


## Experimental

### 

#### Crystal data

C_13_H_4_O_2_S_5_
*M* *_r_* = 352.46
Triclinic, 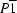

*a* = 7.8527 (8) Å
*b* = 8.0281 (9) Å
*c* = 12.0022 (13) Å
α = 97.934 (9)°
β = 89.227 (9)°
γ = 117.867 (8)°
*V* = 661.37 (12) Å^3^
*Z* = 2
Mo *K*α radiation
μ = 0.87 mm^−1^
*T* = 296 K
0.48 × 0.12 × 0.08 mm

#### Data collection

Siemens P4 diffractometer
Absorption correction: ψ scan (*XSCANS*; Siemens, 1996 ▶) *T*_min_ = 0.679, *T*_max_ = 0.733
3881 measured reflections
2323 independent reflections
1748 reflections with *I* > 2σ(*I*)
*R*_int_ = 0.026
2 standard reflections every 48 reflections  intensity decay: 2%

#### Refinement

*R*[*F* ^2^ > 2σ(*F* ^2^)] = 0.035
*wR*(*F* ^2^) = 0.090
*S* = 1.02
2323 reflections
181 parameters
H-atom parameters constrained
Δρ_max_ = 0.27 e Å^−3^
Δρ_min_ = −0.28 e Å^−3^

### 

Data collection: *XSCANS* (Siemens, 1996 ▶); cell refinement: *XSCANS*; data reduction: *XSCANS*; program(s) used to solve structure: *SHELXS97* (Sheldrick, 2008 ▶); program(s) used to refine structure: *SHELXL97* (Sheldrick, 2008 ▶); molecular graphics: *Mercury* (Macrae *et al.*, 2006 ▶); software used to prepare material for publication: *SHELXL97*.

## Supplementary Material

Crystal structure: contains datablock(s) I, global. DOI: 10.1107/S1600536811039079/yk2021sup1.cif

Structure factors: contains datablock(s) I. DOI: 10.1107/S1600536811039079/yk2021Isup2.hkl

Supplementary material file. DOI: 10.1107/S1600536811039079/yk2021Isup3.mol

Supplementary material file. DOI: 10.1107/S1600536811039079/yk2021Isup4.cml

Additional supplementary materials:  crystallographic information; 3D view; checkCIF report

## Figures and Tables

**Fig. 1.**
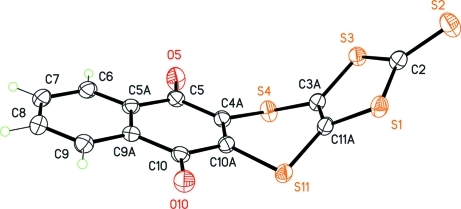
The structure of the title compound, with displacement ellipsoids for non-H atoms at the 30% probability level.

**Fig. 2.**
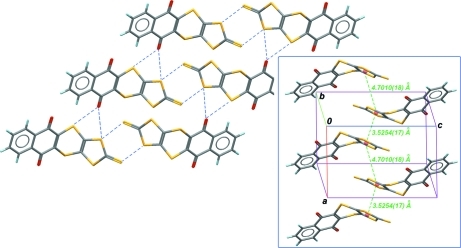
A partial view of the crystal structure for the title compound, with intermolecular S···S and S···O contacts showed as dashed lines. The inset is a view of the crystal normal to the b* axis. Quoted distances are separations between the centroids of neighboring dithiole rings, stacked along the a axis.

## supplementary crystallographic information

### Comment

The development of new types of π-electron donors and acceptors with high polarizability continues to be an attractive topic in material sciences. Such compounds are not only interesting as candidates for single-component conductors, but also because they have low excitation energies and promising applications as NLO materials and near-IR absorbing dyes. Sulfur-based heterocycles are good candidates for building such materials, and donors like TTF and BEDT-TTF became emblematic systems in the 70's, after they allowed to synthesize molecular metals and superconductor materials (Wudl, 1984; Jérome, 2007).

The title compound belongs to the 1,4-dithiine derivatives, which have a particular conformational flexibility, because the energy barrier between the planar and boat conformations is very low (Hayakawa *et al.*, 1982). *Ab initio* computations showed for example that for 1,4-dithiine, the *C*_2_*_v_*(boat) →*D*_2_*_h_*(planar) conformational interconversion requires less than 3 kcal/mol (see Table II and Fig. 4 in Kao *et al.*, 1985). A fine tuning of the geometry and electron distribution may thus be expected by varying the substituents of this heterocycle. For example, electron withdrawing groups seem to stabilize the unfolded conformer (Brisse *et al.*, 2000).

In contrast, the title molecule (Kniess & Mayer, 1996; Mendez-Rojas *et al.*, 2001) adopts a folded conformation (Fig. 1). The dihedral angle between the essentially planar naphthoquinone ring (C4a/C5/C5a/C6···C9/C9a/C10/C10*a*; max. deviation: 0.028 Å for C4a) and the five membered 1,3-dithiole ring (S1/C2/S3/C3a/C11*a*; max. deviation: 0.016 Å for C2) is 47.83 (6)°. This boat conformation is favored by intramolecular S···O repulsion effects, characterized by non-bonding distances S11···O10 = 2.874 (2) and S4···O5 = 2.868 (2) Å. Heteroatoms are also involved in intermolecular contacts. Molecules form centrosymmetric dimers through S1···S2*^i^* contacts [3.5761 (13) Å; symmetry code (*i*): 1 - *x*, 3 - *y*, 1 - *z*] between the thioxo group and one S atom of the dithiole heterocycle. The contacts pattern is completed by bifurcated S1/S11···O5*^ii^* interactions [3.158 (2) and 3.159 (2) Å; symmetry code (*ii*): 1 + *x*, 1 + *y*, *z*], forming a two-dimensional network of contacts in the crystal (Fig. 2). This arrangement is compatible with a stacking structure for molecules, in the [100] direction: two dithiole rings related by inversion give a π···π interaction characterized by a centroid to centroid separation of 3.5254 (17) Å. However, as a consequence of the triclinic symmetry, the following stacked ring generated by inversion is found at a different distance, 4.7010 (18) Å. Short and long separations thus alternate along the stack (Fig. 2, inset), a common situation for one-dimensional materials affected by a Peierls distortion.

The title molecule is isomorphous with the 2-oxo analogue (Mendez-Rojas *et al.*, 2001). However, it is interesting to note that both the molecular and the crystal structures present significantly different metrics for the 2-thioxo and the 2-oxo compounds. In the latter, the folding angle is 42.3 (1)°, and the separations in the dithiole stacks parallel to [100] are 3.566 and 4.345 Å. Despite of the clear dimerization along the stacks for both compounds, the title molecule seems to be more prone to Peierls instability, compared to its 2-oxo analogue.

### Experimental

The precursor (NBu_4_)_2_[Zn(*dmit*)_2_], where H_2_*dmit* is 4,5-dimercapto-1,3-dithiole-2-thione, was prepared as previously reported (Wang *et al.*, 1998). This complex (1.68 g, 2.20 mmol in 20 ml acetone) was reacted with 2,3-dichloro-1,4-naphthoquinone (1 g, 4.40 mmol) at room temperature, forming immediately a dark precipitate. The mixture was stirred overnight and the precipitate was then recovered by vacuum filtration (1.50 g, 99%) and recrystallized from CH_2_Cl_2_. Small black shiny needles suitable for X-ray diffraction were obtained after several days. *M*.p. 354–355 °C. IR (KBr, cm^-1^) 1663 (*vs*), 1586 (*m*), 1553 (*m*), 1493 (*m*), 1385 (sm), 1275 (*vs*), 1134 (*m*), 1073 (*vs*), 794 (*m*), 706 (*s*), 635 (sm), 505 (sm); ^1^H-NMR (CDCl_3_, p.p.m.) δ, 7.80 (dd, 2H), 8.16 (dd, 2H). Anal. calcd. for C_13_H_4_O_2_S_5_: C 44.3%; found: C 42.9%.

### Refinement

The four aromatic H atoms of the naphthoquinone were placed in idealized positions and refined with C—H bond lengths fixed to 0.93 Å and isotropic displacement parameters fixed to 1.2 times the equivalent displacement of the carrier C atom.

### Crystal data

**Table:**

C_13_H_4_O_2_S_5_	*Z* = 2
*M**_r_* = 352.46	*F*(000) = 356
Triclinic, *P*1	*D*_x_ = 1.770 Mg m^−^^3^
Hall symbol: -P 1	Melting point: 627 K
*a* = 7.8527 (8) Å	Mo *K*α radiation, λ = 0.71073 Å
*b* = 8.0281 (9) Å	Cell parameters from 68 reflections
*c* = 12.0022 (13) Å	θ = 4.8–12.3°
α = 97.934 (9)°	µ = 0.87 mm^−^^1^
β = 89.227 (9)°	*T* = 296 K
γ = 117.867 (8)°	Needle, brown
*V* = 661.37 (12) Å^3^	0.48 × 0.12 × 0.08 mm

### Data collection

**Table:**

Siemens P4 diffractometer	1748 reflections with *I* > 2σ(*I*)
Radiation source: fine-focus sealed tube	*R*_int_ = 0.026
graphite	θ_max_ = 25.1°, θ_min_ = 2.9°
ω scans	*h* = −5→8
Absorption correction: ψ scan (*XSCANS*; Siemens, 1996)	*k* = −9→8
*T*_min_ = 0.679, *T*_max_ = 0.733	*l* = −14→14
3881 measured reflections	2 standard reflections every 48 reflections
2323 independent reflections	intensity decay: 2%

### Refinement

**Table:**

Refinement on *F*^2^	Primary atom site location: structure-invariant direct methods
Least-squares matrix: full	Secondary atom site location: difference Fourier map
*R*[*F*^2^ > 2σ(*F*^2^)] = 0.035	Hydrogen site location: inferred from neighbouring sites
*wR*(*F*^2^) = 0.090	H-atom parameters constrained
*S* = 1.02	*w* = 1/[σ^2^(*F*_o_^2^) + (0.0433*P*)^2^ + 0.1487*P*] where *P* = (*F*_o_^2^ + 2*F*_c_^2^)/3
2323 reflections	(Δ/σ)_max_ < 0.001
181 parameters	Δρ_max_ = 0.27 e Å^−^^3^
0 restraints	Δρ_min_ = −0.28 e Å^−^^3^
0 constraints	

### Fractional atomic coordinates and isotropic or equivalent isotropic displacement parameters (Å^2^)

**Table:**

	*x*	*y*	*z*	*U*_iso_*/*U*_eq_	
S1	0.44687 (11)	1.23625 (11)	0.59078 (6)	0.0435 (2)	
C2	0.3040 (4)	1.1337 (4)	0.4656 (2)	0.0430 (7)	
S2	0.30632 (14)	1.25421 (13)	0.36621 (8)	0.0631 (3)	
S3	0.15541 (11)	0.88891 (11)	0.45820 (6)	0.0439 (2)	
C3A	0.2361 (4)	0.8652 (4)	0.5872 (2)	0.0375 (7)	
S4	0.12380 (12)	0.64220 (10)	0.63387 (6)	0.0463 (2)	
C4A	0.1022 (4)	0.7202 (4)	0.7760 (2)	0.0369 (7)	
C5	−0.0767 (4)	0.5886 (4)	0.8258 (2)	0.0383 (7)	
O5	−0.1952 (3)	0.4454 (3)	0.76730 (18)	0.0604 (7)	
C5A	−0.0991 (4)	0.6353 (4)	0.9471 (2)	0.0372 (7)	
C6	−0.2599 (4)	0.5131 (4)	0.9978 (3)	0.0459 (8)	
H6A	−0.3551	0.4037	0.9552	0.055*	
C7	−0.2798 (4)	0.5531 (5)	1.1123 (3)	0.0525 (9)	
H7A	−0.3879	0.4708	1.1464	0.063*	
C8	−0.1369 (4)	0.7168 (5)	1.1754 (3)	0.0521 (9)	
H8A	−0.1500	0.7437	1.2519	0.063*	
C9	0.0245 (4)	0.8404 (5)	1.1260 (2)	0.0443 (7)	
H9A	0.1192	0.9495	1.1692	0.053*	
C9A	0.0449 (4)	0.8014 (4)	1.0116 (2)	0.0363 (7)	
C10	0.2197 (4)	0.9333 (4)	0.9599 (2)	0.0364 (7)	
O10	0.3465 (3)	1.0782 (3)	1.01299 (16)	0.0490 (6)	
C10A	0.2389 (4)	0.8801 (4)	0.8376 (2)	0.0353 (6)	
S11	0.45668 (10)	1.03981 (11)	0.78538 (6)	0.0444 (2)	
C11A	0.3701 (4)	1.0256 (4)	0.6482 (2)	0.0376 (7)	

### Atomic displacement parameters (Å^2^)

**Table:**

	*U*^11^	*U*^22^	*U*^33^	*U*^12^	*U*^13^	*U*^23^
S1	0.0400 (4)	0.0385 (4)	0.0406 (4)	0.0090 (3)	0.0052 (3)	0.0064 (3)
C2	0.0377 (16)	0.0456 (17)	0.0435 (16)	0.0176 (14)	0.0072 (13)	0.0073 (14)
S2	0.0727 (6)	0.0530 (5)	0.0559 (5)	0.0202 (5)	−0.0066 (4)	0.0183 (4)
S3	0.0421 (4)	0.0420 (4)	0.0388 (4)	0.0127 (3)	−0.0030 (3)	0.0046 (3)
C3A	0.0347 (15)	0.0379 (15)	0.0363 (15)	0.0146 (13)	0.0046 (12)	0.0039 (12)
S4	0.0571 (5)	0.0334 (4)	0.0363 (4)	0.0125 (4)	0.0006 (3)	0.0004 (3)
C4A	0.0337 (15)	0.0350 (15)	0.0354 (15)	0.0105 (13)	−0.0030 (12)	0.0054 (12)
C5	0.0325 (15)	0.0316 (15)	0.0408 (15)	0.0062 (13)	−0.0062 (12)	0.0061 (12)
O5	0.0494 (13)	0.0469 (13)	0.0490 (13)	−0.0065 (11)	−0.0059 (11)	0.0038 (11)
C5A	0.0288 (15)	0.0384 (15)	0.0416 (15)	0.0124 (12)	−0.0029 (12)	0.0092 (12)
C6	0.0318 (16)	0.0468 (18)	0.0549 (19)	0.0128 (14)	0.0009 (14)	0.0160 (14)
C7	0.0346 (17)	0.066 (2)	0.059 (2)	0.0211 (17)	0.0104 (15)	0.0259 (17)
C8	0.0460 (19)	0.075 (2)	0.0421 (17)	0.0331 (18)	0.0108 (15)	0.0153 (16)
C9	0.0414 (17)	0.0533 (19)	0.0381 (16)	0.0228 (15)	−0.0007 (13)	0.0037 (14)
C9A	0.0323 (15)	0.0418 (16)	0.0362 (15)	0.0179 (13)	−0.0013 (12)	0.0080 (12)
C10	0.0333 (15)	0.0379 (16)	0.0350 (15)	0.0144 (13)	−0.0056 (12)	0.0047 (12)
O10	0.0412 (12)	0.0455 (12)	0.0388 (11)	0.0047 (10)	−0.0066 (9)	−0.0021 (9)
C10A	0.0302 (15)	0.0337 (15)	0.0349 (14)	0.0089 (12)	−0.0003 (12)	0.0057 (12)
S11	0.0287 (4)	0.0493 (5)	0.0367 (4)	0.0032 (3)	−0.0019 (3)	0.0060 (3)
C11A	0.0327 (15)	0.0392 (16)	0.0349 (14)	0.0122 (13)	0.0048 (12)	0.0053 (12)

### Geometric parameters (Å, °)

**Table:**

S1—C2	1.742 (3)	C6—C7	1.392 (4)
S1—C11A	1.748 (3)	C6—H6A	0.9300
C2—S2	1.631 (3)	C7—C8	1.389 (5)
C2—S3	1.744 (3)	C7—H7A	0.9300
S3—C3A	1.747 (3)	C8—C9	1.381 (4)
C3A—C11A	1.340 (4)	C8—H8A	0.9300
C3A—S4	1.756 (3)	C9—C9A	1.389 (4)
S4—C4A	1.768 (3)	C9—H9A	0.9300
C4A—C10A	1.349 (4)	C9A—C10	1.480 (4)
C4A—C5	1.487 (4)	C10—O10	1.216 (3)
C5—O5	1.215 (3)	C10—C10A	1.497 (4)
C5—C5A	1.482 (4)	C10A—S11	1.762 (3)
C5A—C6	1.383 (4)	S11—C11A	1.758 (3)
C5A—C9A	1.409 (4)		
			
C2—S1—C11A	96.73 (14)	C8—C7—H7A	120.3
S2—C2—S1	123.61 (18)	C6—C7—H7A	120.3
S2—C2—S3	123.38 (18)	C9—C8—C7	120.9 (3)
S1—C2—S3	113.00 (17)	C9—C8—H8A	119.5
C2—S3—C3A	96.94 (14)	C7—C8—H8A	119.5
C11A—C3A—S3	116.4 (2)	C8—C9—C9A	119.8 (3)
C11A—C3A—S4	124.4 (2)	C8—C9—H9A	120.1
S3—C3A—S4	118.94 (16)	C9A—C9—H9A	120.1
C3A—S4—C4A	98.37 (13)	C9—C9A—C5A	119.6 (3)
C10A—C4A—C5	121.6 (3)	C9—C9A—C10	119.4 (3)
C10A—C4A—S4	123.9 (2)	C5A—C9A—C10	121.0 (2)
C5—C4A—S4	114.4 (2)	O10—C10—C9A	122.8 (2)
O5—C5—C5A	122.6 (3)	O10—C10—C10A	120.0 (2)
O5—C5—C4A	119.4 (3)	C9A—C10—C10A	117.3 (2)
C5A—C5—C4A	118.0 (2)	C4A—C10A—C10	121.8 (2)
C6—C5A—C9A	119.9 (3)	C4A—C10A—S11	124.2 (2)
C6—C5A—C5	119.9 (3)	C10—C10A—S11	113.92 (19)
C9A—C5A—C5	120.3 (2)	C11A—S11—C10A	98.74 (13)
C5A—C6—C7	120.3 (3)	C3A—C11A—S1	116.9 (2)
C5A—C6—H6A	119.8	C3A—C11A—S11	124.3 (2)
C7—C6—H6A	119.8	S1—C11A—S11	118.53 (16)
C8—C7—C6	119.5 (3)		
			
C11A—S1—C2—S2	−178.7 (2)	C5—C5A—C9A—C9	−178.1 (3)
C11A—S1—C2—S3	2.07 (19)	C6—C5A—C9A—C10	179.2 (3)
S2—C2—S3—C3A	178.6 (2)	C5—C5A—C9A—C10	0.8 (4)
S1—C2—S3—C3A	−2.19 (19)	C9—C9A—C10—O10	−1.4 (4)
C2—S3—C3A—C11A	1.5 (3)	C5A—C9A—C10—O10	179.7 (3)
C2—S3—C3A—S4	175.85 (18)	C9—C9A—C10—C10A	177.3 (3)
C11A—C3A—S4—C4A	38.1 (3)	C5A—C9A—C10—C10A	−1.6 (4)
S3—C3A—S4—C4A	−135.80 (18)	C5—C4A—C10A—C10	0.7 (4)
C3A—S4—C4A—C10A	−39.0 (3)	S4—C4A—C10A—C10	−175.8 (2)
C3A—S4—C4A—C5	144.2 (2)	C5—C4A—C10A—S11	178.5 (2)
C10A—C4A—C5—O5	−179.5 (3)	S4—C4A—C10A—S11	1.9 (4)
S4—C4A—C5—O5	−2.6 (4)	O10—C10—C10A—C4A	179.6 (3)
C10A—C4A—C5—C5A	−1.6 (4)	C9A—C10—C10A—C4A	0.9 (4)
S4—C4A—C5—C5A	175.3 (2)	O10—C10—C10A—S11	1.6 (4)
O5—C5—C5A—C6	0.2 (5)	C9A—C10—C10A—S11	−177.1 (2)
C4A—C5—C5A—C6	−177.6 (3)	C4A—C10A—S11—C11A	36.5 (3)
O5—C5—C5A—C9A	178.6 (3)	C10—C10A—S11—C11A	−145.6 (2)
C4A—C5—C5A—C9A	0.8 (4)	S3—C3A—C11A—S1	−0.3 (3)
C9A—C5A—C6—C7	−0.1 (5)	S4—C3A—C11A—S1	−174.26 (16)
C5—C5A—C6—C7	178.3 (3)	S3—C3A—C11A—S11	174.17 (16)
C5A—C6—C7—C8	0.0 (5)	S4—C3A—C11A—S11	0.2 (4)
C6—C7—C8—C9	0.0 (5)	C2—S1—C11A—C3A	−1.1 (3)
C7—C8—C9—C9A	0.1 (5)	C2—S1—C11A—S11	−175.89 (18)
C8—C9—C9A—C5A	−0.2 (5)	C10A—S11—C11A—C3A	−37.8 (3)
C8—C9—C9A—C10	−179.1 (3)	C10A—S11—C11A—S1	136.56 (18)
C6—C5A—C9A—C9	0.3 (4)		

## References

[bb1] Brisse, F., Atfani, M., Bergeron, J.-Y., Bélanger-Gariépy, F. & Armand, M. (2000). *Acta Cryst.* C**56**, 190–192. 10.1107/s0108270199010100 10777883

[bb2] Hayakawa, K., Mibu, N., Ōsawa, E. & Kanematsu, K. (1982). *J. Am. Chem. Soc.* **104**, 7136–7142.

[bb3] Jérome, D. (2007). *Physics of organic superconductors and conductors*, edited by A. G. Lebed, pp. 3–16. Berlin: Springer.

[bb4] Kao, J., Eyermann, C., Southwick, E. & Leister, D. (1985). *J. Am. Chem. Soc.* **107**, 5323–5332.

[bb5] Kniess, T. & Mayer, R. (1996). *Z. Naturforsch. Teil B*, **51**, 901–904.

[bb6] Macrae, C. F., Edgington, P. R., McCabe, P., Pidcock, E., Shields, G. P., Taylor, R., Towler, M. & van de Streek, J. (2006). *J. Appl. Cryst.* **39**, 453–457.

[bb7] Mendez-Rojas, M. A., Bodige, S. G., Ejsmont, K. & Watson, W. H. (2001). *J. Chem. Crystallogr.* **31**, 17–28.

[bb8] Sheldrick, G. M. (2008). *Acta Cryst.* A**64**, 112–122. 10.1107/S0108767307043930 18156677

[bb9] Siemens (1996). *XSCANS* Siemens Analytical X-ray Instruments Inc., Madison, Wisconsin, USA.

[bb10] Wang, C. S., Batsanov, A. S., Bryce, M. R. & Howard, J. A. K. (1998). *Synthesis*, pp. 1615–1618.

[bb11] Wudl, F. (1984). *Acc. Chem. Res.* **17**, 227–232.

